# Maternal depression and adolescent optimism

**DOI:** 10.1016/j.ssmph.2022.101135

**Published:** 2022-06-24

**Authors:** Jessica Halliday Hardie, Kristin Turney

**Affiliations:** a Department of Sociology, Hunter College and the Graduate Center, CUNY, 695 Park Avenue, 16th Floor Hunter West, New York, NY, 10065, USA; b Department of Sociology, University of California, 3151 Social Science Plaza, Irvine, CA, 92697-5100, USA

**Keywords:** Life course, Parenting, Depression, Adolescents, Optimism

## Abstract

The life course perspective posits that parents' and children's lives are linked through shared experiences and interdependent contexts such as the household. In this paper, we draw on the life course perspective to examine the relationship between maternal depression and adolescent optimism, an important trait that reflects adolescents' positive expectations for the future, and how features of the family context explain this association. We use data from the Fragile Families and Child Wellbeing Study (N = 3013), taking advantage of the study's longitudinal measures of maternal depression that span a 15-year period. First, we find that current maternal depression is negatively associated with optimism among adolescents. Second, we find that the family environment and parent-child relationships, but not economic wellbeing, explain the association between maternal depression and adolescent optimism. These findings inform our understanding of how parent and adolescent wellbeing are linked and, importantly, how the family environment conditions how adolescents envision their futures.

## Highlights

• Current maternal depression is negatively associated with optimism among adolescents.
• Family environment and parent-child relationships are mediators in this relationship.
• Economic wellbeing does not mediate the relationship between maternal depression and adolescent optimism.

## 1 Introduction

An estimated 15.6 million children in the United States live with an adult who endured a major depressive episode in the prior year (England & Sim, 2009), and the prevalence of depression and depressive symptoms is higher among adults with lower socioeconomic status compared to adults with higher socioeconomic status (Oh, Salas-Wright, & Vaughn, 2018; Todd & Teitler, 2019). Research suggests that these periods of depression, particularly when experienced by mothers, can critically impair their children's wellbeing (Cummings, Cheung, Koss, & Davies, 2014; Ehrlich, Chen, Yu, Miller, & Brody, 2019; Livings, 2021). Children and adolescents whose mothers experience depression, for example, struggle with internalizing and externalizing behavior problems more than their counterparts (Kiernan & Huerta, 2008; Turney, 2011a). These youth also have higher risk for depression and other mental health problems, physical health conditions, and premature mortality (Goodman & Gotlib, 1999; Turney, 2011b; Weissman et al., 2016).

Research shows that maternal depression increases or intensifies *negative* outcomes for children, but less is known about whether or how these repercussions extend to adolescents or to indicators of adolescent wellbeing such as optimism. Optimism is an important trait that reflects adolescents' positive expectations for the future and is associated with a range of outcomes including better physical health, proactive health-seeking behaviors, educational persistence, and romantic relationship quality (Alarcon, Bowling, & Khazon, 2013; Carver, Scheier, & Segerstrom, 2010). Maternal depression may be negatively associated with optimism among adolescents. This association may operate directly, because mothers' own pessimistic view of the future influences their offspring's expectations, or indirectly, due to how maternal depression can alter the home environment and family relationships (Lovejoy, Graczyk, O’Hare, & Neuman, 2000; Reichman, Corman, & Noonan, 2015; Turney & Hardie, 2021; Williams, 2018), which in turn may have damaging repercussions for adolescent optimism. Alternatively, maternal depression may be spurious after accounting for factors associated with selection into experiencing maternal depression such as socioeconomic status (Dooley, Prause, & Ham-Rowbottom, 2000; Lorant et al., 2003). The timing of maternal depression may also matter, with mothers' prior depression setting children on a path toward lower optimism in adolescence or with mothers' depression having a more concurrent association with adolescent optimism (Turney, 2011a).

In this study, we contribute to research on families, health, and inequality by using data from the Fragile Families and Child Wellbeing Study to examine the relationships between current and prior maternal depression and adolescent optimism, accounting for a range of confounding factors. We also examine three mechanisms underlying these relationships: family environment, parent-adolescent relationships, and economic wellbeing. Our analyses, grounded in the life course perspective (Elder, 1998), demonstrate how one family member's mental health problems can reverberate into family life, having consequences for adolescent optimism. Although prior research has demonstrated associations between maternal depression and a range of children's outcomes, including emotion regulation and depression (Goodman et al., 2011), our study advances the literature by considering how maternal depression shapes adolescent offsprings' positive outlook about the future, whether this association holds for both current and prior maternal depression, and the role of family contexts in explaining this association. Our findings contribute to an understanding of how the life course trajectories of family members are interconnected (Bronfenbrenner, 1977) and how maternal mental health promotes positive adolescent functioning.

## 2 Background

### 2.1 Importance of optimism among adolescents

Adolescence is a key life course period in which young people begin to develop their sense of self in relation to others and the wider world (Steinberg & Morris, 2001). Young people's positive wellbeing during this period is critical; it shapes the kinds of behaviors they choose to engage in, their plans for the future, and their ability to navigate stressful situations (Larson, 2000; Park, 2004). In particular, possessing a positive future orientation is important for adolescents' ability to envision themselves in the future, and it is linked to forging clear goals for the future and strong planning (Johnson, Blum, & Cheng, 2014). In this paper, we focus on optimism, a construct of positive future orientation that represents one's usual feelings of hope or positive expectations for what will occur in the future. We focus on this outcome because it is fairly broad—that is, optimism as a trait can be applied to a wide variety of contexts—and is associated with a number of positive outcomes for adolescents (Johnson et al., 2014).

### 2.2 Linking maternal depression to adolescent optimism

The life course perspective posits that family members' wellbeing is interconnected through family processes in which setbacks or opportunities for one family member have repercussions for others in the family (Elder, 1998). In other words, events that initially impact just one family member can reverberate to other family members through a series of family processes, including interpersonal relationships, the rearrangement of family resources, and alterations in daily routines. Research has demonstrated this dynamic among couples facing financial hardship (Hardie & Lucas, 2010; Yeung & Hofferth, 1998), families following a job loss (Brand & Thomas, 2014; Schneider, Harknett, & McLanahan, 2016), children whose parents report health problems (Hardie & Turney, 2017), and families experiencing paternal incarceration (Turney, 2017).

Furthermore, the life course perspective argues that “the developmental impact of a succession of life transitions or events is contingent on when they occur in a person's life,” (Elder, 1998, p. 3). For example, the Great Depression had different—and even opposite—associations with young people's life trajectories depending on the timing of this event relative to their age (Elder, 1974; Elder, Liker, & Jaworski, 2013). Prior research finds that the repercussions of living in particular family and household conexts vary based on timing. For example, some research suggests that exposure to poverty in early childhood can have more severe detrimental consequences for achievement than exposure to poverty in later childhood and adolescence (Duncan, Yeung, Brooks-Gunn, & Smith, 1998). Other research similarly finds that experiences of paternal incarceration are especially detrimental in early childhood (Turney, 2021).

In this paper, we examine whether maternal depression is associated with adolescent optimism. Depression affects one's mood, energy, engagement with others, health behaviors, and self-care (Clark & Watson, 1991; Katon, 2003; Oquendo et al., 2004), and these changes can manifest in parents' daily functioning and interactions with their children. For example, prior research demonstrates that maternal depression structures children's mental and physical health, access to health care, and parenting practices (Goodman et al., 2011; Minkovitz et al., 2005; Turney, 2011a, 2011b; Wickramaratne et al., 2011). Our research complements and expands this work by examining how maternal depression is associated with optimism, whether this association varies by the timing of maternal depression, and whether household and family contexts explain this association. Although adolescents are less dependent upon their parents than young children, this developmental stage is associated with changing parent-child relationships and elevated parent-child conflict (Steinberg & Morris, 2001). Maternal depression may intensify or disrupt this developmental process and young people's positive outlook about the future. Furthermore, research on family-based interventions to alleviate the impact of parental depression on children's outcomes have shown that these interventions work well when children are in late childhood and early adolescence, when clinicians can target both parents' symptoms and children's coping mechanisms (Compas et al., 2009).

### 2.3 Mechanisms linking maternal depression and adolescent optimism

We posit three mechanisms through which maternal depression may shape adolescent optimism: family environment, parent-adolescent relationship, and economic wellbeing. First, the family environment may explain the association between maternal depression and adolescent optimism. We define family environment as consisting of the structural aspects of the home: the presence or absence of romantic partners, the quality of the parental relationship, and daily household characteristics such as noise and patterns of behavior. Depression is associated with marital conflict (Cummings et al., 2014), which can lead to relationship dissolution and more relationship churning (Meadows, McLanahan, & Brooks-Gunn, 2008). Depression is also characterized by an inability to complete daily tasks (van Wijngaarden, Schene, & Koeter, 2004), which can engender greater household disruptions. In turn, poor parental relationships, family structure disruptions, and a chaotic family environment may impair adolescents' optimism about the future (Franklin, Janoff-Bulman, & Roberts, 1990; Orejudo, Puyuelo, Fernández-Turrado, & Ramos, 2012).

Second, weakened mother-child relationships may explain the association between maternal depression and adolescent optimism. Depression affects one's mood, communicativeness, and hostility (Burke, 2003; Clark & Watson, 1991; Perlis et al., 2005), which can spillover into the mother-child relationship. Mothers may withdraw from interactions with their children or lash out due to frustration. Mothers may also experience a greater degree of stress when coping with both depression and the demands of parenting. Even when depression occurred in the past, it may continue to impair mother-child relationships over time. In turn, poor mother-child relationships are associated with negative outcomes for children and youth (Laursen & Collins, 2009; Steinberg, 2001). One study found that optimism was positively associated with parent-child communication and negatively associated with parent-child disagreements (Orejudo et al., 2012).

Third, family socioeconomic status may explain the association between maternal depression and adolescent optimism. Depression is associated with a lower likelihood of holding a job, fewer work hours, and less satisfactory job performance (Dagher, Hofferth, & Lee, 2014; Lerner et al., 2004; Lerner & Henke, 2008). These difficulties maintaining stable employment can impair household finances and increase material hardship, particularly for mothers in already low-income households (Garg, Toy, Tripodis, Cook, & Cordella, 2015; Morrissey, Cha, Wolf, & Khan, 2020). Depression may also cost money to treat, or may spur poor financial decisions that drain finances. In turn, concerns over money may reduce adolescent optimism directly, through limitations on the kinds of activities they can participate in or resources they have available, or indirectly, through concern over money and long-term stability. Indeed, parental socioeconomic status in childhood is associated with young adult optimism, even after accounting for the young adult's own socioeconomic status (Heinonen et al., 2006).

### 2.4 Potential confounders in the association between maternal depression and adolescent optimism

There are a number of factors that are associated with both maternal depression and adolescent optimism and therefore may act as confounders. Experiences of discrimination on the basis of racial/ethnic background or immigrant status may trigger depression in mothers and independently be associated with young people's optimism (Schulz et al., 2006; Torres & Ong, 2010). Research also demonstrates that poverty and material hardship are linked to depression such that early experiences of economic need trigger depressive symptoms (Belle & Doucet, 2003; Heflin & Iceland, 2009; Ridley, Rao, Schilbach, & Patel, 2020; Williams & Cheadle, 2016). Exposure to stressful life events, including experiences of and related to poverty, is also associated with lower levels of optimism and hope among young people (Gillham & Reivich, 2004; Otis, Huebner, & Hills, 2016). Dynamics internal to family life, such as family structure, romantic relationship quality, and household composition, can exacerbate or mitigate daily stress for both mothers and children (Osborne, Berger, & Magnuson, 2012; Rasmussen et al., 2022). Mother's age and social support from others are also associated with depression, serve as potential resources for coping when stressful situations arise, and can affect parenting styles that influence optimism (Gjesfjeld, Greeno, Kim, & Anderson, 2010; Wang, Wu, Anderson, & Florence, 2011). Finally, children's age, temperament, and gender are associated with youth optimism and maternal experiences with parenting (Otis et al., 2016).

## 3 Material and methods

### 3.1 Data

This paper examines the relationship between current and prior maternal depression and adolescent optimism. We use data from the Fragile Families and Child Wellbeing Study, a longitudinal survey of 4898 children born to parents living in urban areas between 1998 and 2000 (Reichman, Teitler, Garfinkel, & McLanahan, 2001). The research design oversampled for unmarried parents and, accordingly, about three-fourths of parents were unmarried. All mothers and about two-thirds (66%) of fathers were first interviewed in hospitals, as soon as possible after their child's birth, and parents were re-interviewed by telephone when children were 1, 3, 5, 9, and 15 years old (with only the child's primary caregiver being interviewed at the 15-year survey). Children were also interviewed when they were 9 and 15 years old.

The analytic sample includes 3013 observations. We exclude 1454 adolescents who did not participate in the 15-year survey and an additional 7 adolescents missing information on the dependent variable (described below). We also exclude 424 adolescents who had a non-maternal caregiver participate in the 15-year survey. Comparisons of baseline characteristics between the analytic and full samples show some small differences between these samples. Compared to mothers in the full sample, mothers in the analytic sample are more likely to be non-Hispanic Black (50% compared to 48%, *p* < .05), less likely to be Hispanic (25% compared to 27%, *p* < .05), and less likely to be foreign-born (14% compared to 17%, *p* < .001). Mothers in the analytic sample are also less likely to have less than a high school diploma (31% compared to 35%, *p* < .001) and are more likely to report employment plans (71% compared to 68%, *p* < .01).

### 3.2 Measures

*3.2.1. Adolescent Optimism*. The dependent variable, adolescent optimism, was drawn from the EPOCH Measure of Adolescent Wellbeing (Kern, Benson, Steinberg, & Steinberg, 2016). Adolescents were asked to respond to the following four statements (1 = *strongly disagree* to 4 = *strongly agree*): (a) I am optimistic about my future; (b) I think good things are going to happen to me; (c) I believe that things will work out, no matter how difficult they seem; and (d) in uncertain times, I expect the best (α = 0.55). Although this Cronbach's alpha is relatively low, this measure has been validated with the Fragile Families data using confirmatory factor analysis (Choi, Ryu, & Yang, 2021). Higher values indicate more optimism.

*3.2.2. Maternal Depression*. At the 1-, 3-, 5-, 9-, and 15-year surveys, maternal depression was measured based on responses to the Composite International Diagnostic Interview Short Form (CIDI-SF), Version 1.0, November 1998 (Kessler, Andrews, Mroczek, Ustun, & Wittchen, 1998), an instrument commonly used in large-scale surveys to measure major depressive disorder (MDD) (Aalto-Setälä et al., 2002). Mothers were first asked whether, at some time during the previous year, they had feelings of depression or were unable to enjoy things they normally found pleasurable. Those who experienced at least one of these two conditions most of the day, every day, for a two-week period were asked to report on the following: (a) losing interest in things; (b) feeling tired; (c) experiencing a weight change of at least 10 pounds; (d) having trouble sleeping; (e) having trouble concentrating; (f) feeling worthless; and (g) thinking about death. Those who answered affirmatively to at least one of the stem questions and three of the additional questions are considered as likely having MDD in the previous year. At the 1-, 3-, 5-, and 9-year surveys, no additional questions were asked about depressive symptoms occurring more than a year before. At the 15-year survey, mothers were asked to report on both the past year and on the past six years in separate questions. We coded anyone who indicated that they had experienced depression in the year preceding the 15-year survey as having current depression. Anyone who reported depression at the 1-, 3-, 5-, or 9-year surveys and anyone who reported depression in the five years prior to the survey year (child ages 9–14), but not the current year (age 15), were included in the prior depression category. Mothers who did not report depression at any survey year were in the no depression group. About half of adolescents had a mother who experienced depression at some point during their childhood (with 18.0% of mothers reporting current depression and 30.3% of mothers reporting prior depression).

#### *3.2.1* *Mechanisms*

We consider three mechanisms that may explain the relationship between maternal depression and adolescent optimism, all measured at the 15-year survey: (1) family environment, (2) parent-adolescent relationship, and (3) economic wellbeing.

First, the family environment was measured with four variables. Separation was measured with a binary variable indicating the adolescent's biological mother and father are not in a romantic relationship. Parent relationship quality was measured by adolescent reports of the relationship quality between his/her parents (1 = *poor* to 5 = *excellent*). Environmental confusion was measured by averaging adolescent reports to the following five statements (1 = *not true* to 3 = *often true*): (a) you can't hear yourself think in your home; (b) it's a real zoo in your home; (c) the children have a regular bedtime routine (reverse coded); (d) you are usually able to stay on top of things (reverse coded); and (e) the atmosphere in your house is calm (reverse coded) (α = .48). Parenting stress was measured by averaging mother responses to the following four statements (1 = *strongly disagree* to 4 = *strongly agree*): (a) being a parent or guardian is harder than I thought it would be; (b) I feel trapped by my responsibilities as a parent or guardian; (c) I find that taking care of my child(ren) is much more work than pleasure; and (d) I often feel tired, worn out, or exhausted from raising a family (α = 0.68).

Second, the parent-adolescent relationship was measured with five variables. Psychological aggression is a binary variable indicating the adolescent reported the mother sometimes or often shouted, yelled, screamed, swore, or cursed at him/her in the past year. Physical aggression is a binary variable indicating the adolescent reported the mother sometimes or often hit or slapped him/her in the past year. Adolescents reported on how close they felt to their mother (1 = *not very close* to 4 = *extremely close*) and father (1 = *not very close* to 4 = *extremely close*). Adolescents also reported on parental monitoring, measured by averaging responses to the following two statements (1 = *never* to 3 = *often*): (a) how often primary caregiver knows what you do during your free time and (b) how often primary caregiver knows what you spend money on (α = 0.59).

Third, economic wellbeing was measured with three variables. A binary measure indicates the mother is employed. A continuous variable captures the log of household income. Material hardship was measured by summing mothers' responses to the following 11 statements, all referring to hardship in the past year (1 = *yes*, 0 = *no*): (a) received free food or meals; (b) was very hungry but didn't eat because couldn't afford enough food; (c) did not pay the full amount of rent or mortgage payments; (d) evicted from home or apartment for not paying the rent or mortgage; (e) did not pay the full amount of gas, oil, or electricity bill; (f) gas or electric services ever turned off, or the heating oil company did not deliver oil, because there wasn't enough money to pay the bills; (g) borrowed money from friends or family to pay the bills; (h) moved in with other people even for a little while because of financial problems; (i) stayed in a shelter, abandoned building, an automobile, or any other place not meant for regular housing; (j) anyone in your household needed to see a doctor or go to the hospital but couldn't go because of the cost; and (k) telephone service (mobile or land line) cancelled or disconnected by the telephone company because there wasn't enough money to pay the bill.

#### *3.2.2* *Control variables*

Multivariate analyses adjusted for demographic, socioeconomic, and behavioral characteristics of parents and adolescents. Unless otherwise noted, control variables that do not change over time (such as race/ethnicity) were measured at the baseline survey and other control variables were measured at the 1-year survey (and, accordingly, at or prior to the measurement of maternal depression).

Control variables include parents' race/ethnicity, immigrant status, age, and childhood family structure (1 = *lived with both parents at age 15*). Family characteristics include relationship status (married, cohabiting, non-residential romantic relationship, separated), repartnership (1 = *partnered with someone besides the child's biological mother or father*), relationship quality between child's parents (1 = *poor* to 5 = *excellent*), number of children in the household, and parenting stress (with the measurement similar to at the 15-year survey). Socioeconomic characteristics include educational attainment (less than high school, high school diploma or GED, some college, and college degree), material hardship (with the measurement also similar to at the 15-year survey), employment, income-to-poverty ratio, and perceived social support. We also adjusted for several parent characteristics that might be especially associated with maternal depression and adolescent wellbeing, including binary variables indicating the mother's parent(s) and the father's parent(s) experienced depression (measured at the 3- and 5-year surveys) and continuous variables measuring mother's and father's cognitive skills (measured by the Weschler Adult Intelligence Scale [WAIS] at the 3-year survey). Child characteristics include gender, temperament (reported by the mother at the 1-year survey), and age (a continuous variable at the 15-year survey).

### 3.3 Analytic strategy

The analytic strategy proceeded in two stages. First, we used ordinary least squares (OLS) regression to estimate adolescent optimism as a function of maternal depression. We present both an unadjusted model (that does not include control variables) and an adjusted model (that does include control variables), the latter of which isolates the relationship between maternal depression and optimism. We adjust for characteristics associated with both maternal depression and adolescent optimism.

Second, we examined three mechanisms that might explain the relationship between maternal depression and adolescent optimism: family environment, parent-adolescent relationship, and economic wellbeing. We estimated the relationship between maternal depression and each of the proposed mechanisms (using linear probability models for the binary outcomes and OLS regression for the others), the relationship between each of the proposed mechanisms and adolescent optimism, the relationship between maternal depression and adolescent optimism without the mechanisms, and the relationship between maternal depression and adolescent optimism with the mechanisms (Baron & Kenny, 1986). We also used the Karlson-Holm-Breen (KHB) method to decompose the total effect into direct and indirect effects (Karlson, Holm, & Breen, 2012).

We used multiple imputation to fill in missing values on independent and control variables, pooling results across 20 imputed data sets (Allison, 2001). Diagnostic tests suggest that multicollinearity does not bias the results.

## 4 Results

### 4.1 Sample description

Table 1 presents descriptive statistics of the sample. The majority of mothers were racial/ethnic minorities, with about half (50.3%) identifying as non-Hispanic Black and one-quarter (24.7%) identifying as Hispanic. On average, mothers were 26 years old and fathers were 29 years old at the 1-year survey. About one-third of mothers (33.2%) and fathers (31.2%) reported that at least one of their parents experienced depression. About two-thirds of youth's biological parents were in a romantic relationship at the 1-year survey (with 30.4% in a marital relationship, 26.5% in a cohabiting relationship, and 10.2% in a non-residential romantic relationship). Adolescents were, on average, 15.6 years old at the 15-year survey.

**Table 1:** Descriptive statistics of sample.

	Mean or %	SD
Adolescent optimism (y15)	3.413	(0.490)
Mother depression (y1, y3, y5, y9, y15)		
Current depression	18.0%	
Prior depression	30.3%	
No depression	51.6%	
Mother race/ethnicity (b)		
White (non-Hispanic)	21.2%	
Black (non-Hispanic)	50.3%	
Hispanic	24.7%	
Other race (non-Hispanic)	3.7%	
Mother and father mixed-race couple (b)	14.6%	
Mother immigrant (b)	14.1%	
Mother lived with both parents at age 15 (b)	42.5%	
Mother parent(s) depressed (y3, y5)	33.2%	
Father parent(s) depressed (y3, y5)	31.2%	
Mother age (y1)	26.425	(5.996)
Father age (y1)	28.835	(7.086)
Mother and father relationship status (y1)		
Married	30.4%	
Cohabiting	26.5%	
Non-residential romantic relationship	10.2%	
Separated	32.9%	
Mother repartnered (y1)	11.6%	
Father repartnered (y1)	11.1%	
Mother relationship quality (y1)	3.293	(1.413)
Father relationship quality (y1)	3.578	(1.231)
Mother number of children in household (y1)	2.284	(1.282)
Father number of children in household (y1)	1.663	(1.414)
Mother parenting stress (y1)	2.174	(0.662)
Father parenting stress (y1)	2.092	(0.681)
Mother educational attainment (y1)		
Less than high school	28.1%	
High school diploma or GED	28.1%	
Some college	31.4%	
College degree	12.5%	
Father educational attainment (y1)		
Less than high school	30.4%	
High school diploma or GED	35.2%	
Some college	23.4%	
College degree	11.0%	
Mother employment (y1)	56.1%	
Father employment (y1)	76.4%	
Mother material hardship (y1)	1.142	(1.600)
Father material hardship (y1)	1.029	(1.562)
Mother income-to-poverty ratio (y1)	1.895	(2.254)
Father income-to-poverty ratio (y1)	2.524	(3.130)
Mother perceived social support (y1)	4.105	(1.788)
Father perceived social support (y1)	4.369	(1.778)
Mother cognitive skills (y3)	6.822	(2.653)
Father cognitive skills (y3)	6.481	(2.721)
Adolescent is male (b)	51.0%	
Adolescent prior temperament (y1)	3.412	(0.760)
Adolescent age, years (y15)	15.572	(0.755)
*Mechanisms*		
Mother-reported separation (y15)	67.7%	
Adolescent-reported parent relationship quality (y15)	2.771	(1.491)
Adolescent-reported environmental confusion (y15)	1.493	(0.370)
Mother-reported parenting stress (y15)	2.058	(0.697)
Adolescent-reported psychological aggression (y15)	65.7%	
Adolescent-reported physical aggression (y15)	12.9%	
Adolescent-reported closeness to mother (y15)	3.431	(0.803)
Adolescent-reported closeness to father (y15)	2.335	(1.227)
Adolescent-reported parental monitoring (y15)	2.700	(0.441)
Mother-reported employment (y15)	71.1%	
Mother-reported material hardship (y15)	1.260	(1.791)
Mother-reported household income, logged (y15)	10.544	(1.112)
N	3013	

Notes: b = measured at baseline, y1 = measured at 1-year survey, y3 = measured at 3-year survey, y5 = measured at 5-year survey, y15 = measured at 15-year survey.

### 4.2 Estimating the relationship between maternal depression and adolescent optimism

Table 2 presents estimates from OLS regression models estimating adolescent optimism as a function of maternal depression. Model 1 presents the unadjusted association. Adolescents of mothers with current depression, compared to adolescents of mothers who never report depression, reported less optimism (*B* = −0.047, *p* < .05). Adolescents of mothers with past depression also reported less optimism than those of mothers who never report depression (*B* = −0.044, *p* < .05).

**Table 2:** OLS regression models estimating adolescent optimism as a function of mother's depression.

	Model 1			Model 2		
	*B*	(S.E.)		*B*	(S.E.)	
Mother depression (reference = no depression)						
Current depression	−0.047	(0.024)	*	−0.043	(0.026)	*
Prior depression	−0.044	(0.021)	*	−0.037	(0.022)	^
Mother race/ethnicity (reference = White [non-Hispanic])						
Black (non-Hispanic)				0.154	(0.028)	***
Hispanic				0.061	(0.031)	*
Other race (non-Hispanic)				0.004	(0.055)	
Mother and father mixed-race couple				0.024	(0.027)	
Mother immigrant				−0.075	(0.032)	**
Mother lived with both parents at age 15				−0.021	(0.020)	
Mother parent(s) depressed				−0.015	(0.021)	
Father parent(s) depressed				−0.008	(0.022)	
Mother age				0.023	(0.002)	
Father age				0.000	(0.002)	
Mother and father relationship status (reference = married)						
Cohabiting				−0.017	(0.028)	
Non-residential romantic relationship				−0.027	(0.040)	
Separated				−0.015	(0.039)	
Mother repartnered				0.001	(0.035)	
Father repartnered				0.027	(0.038)	
Mother relationship quality				−0.018	(0.010)	
Father relationship quality				0.027	(0.010)	
Mother number of children in household				0.001	(0.012)	
Father number of children in household				−0.021	(0.012)	
Mother parenting stress				−0.039	(0.015)	^
Father parenting stress				0.005	(0.016)	
Mother educational attainment (reference = less than high school)						
High school diploma or GED				0.001	(0.025)	
Some college				−0.004	(0.027)	
College degree				−0.036	(0.044)	
Father educational attainment (reference = less than high school)						
High school diploma or GED				0.000	(0.024)	
Some college				0.014	(0.028)	
College degree				−0.010	(0.043)	
Mother employment				−0.015	(0.019)	
Father employment				0.022	(0.024)	
Mother material hardship				0.022	(0.010)	
Father material hardship				−0.068	(0.010)	*
Mother income-to-poverty ratio				−0.002	(0.007)	
Father income-to-poverty ratio				0.032	(0.005)	
Mother perceived social support				0.012	(0.006)	
Father perceived social support				0.007	(0.006)	
Mother cognitive skills				0.010	(0.004)	
Father cognitive skills				−0.060	(0.004)	*
Adolescent is male				0.025	(0.018)	
Adolescent prior temperament				0.004	(0.012)	
Adolescent age				0.029	(0.012)	

Constant	3.438			3.100		
N	3013			3013		

Note: Standardized coefficients presented, with standard errors in parentheses. ^ p < .10, *p < .05, **p < .01, ***p < .001.

Model 2 adjusts for control variables, which together reduced the magnitude of the maternal depression coefficients (by 9% for current maternal depression and 16% for past maternal depression). Current maternal depression remained associated with less optimism among adolescents (*B* = −0.043, *p* < .05). The coefficient for past maternal depression was not statistically significant at conventional thresholds (*B* = −0.037, *n.s.*). In this model, the coefficients for current maternal depression and prior maternal depression were not statistically significantly different from one another (*p* = .565), suggesting that any history of maternal depression was associated with less optimism among adolescents.

#### 4.2.1 Supplemental analyses

Research suggests that girls and boys may differentially respond to maternal depression (Livings, 2021). We considered this possibility by estimating subgroup analyses for boys and girls (available upon request). We found that, net of control variables, the magnitude of the association between current maternal depression and optimism was similar for boys (*B* = −0.046, *n*.*s*.) and girls (*B* = −0.041, *n*.*s*.). We also found that prior maternal depression was similarly associated with optimism for boys (*B* = −0.054, *n.s.*) and girls (*B* = −0.022, *n*.*s*.).

### 4.3 Considering mechanisms linking maternal depression to adolescent optimism

We next consider how three mechanisms—family environment, parent-adolescent relationship, and economic wellbeing—explain the relationship between maternal depression and adolescent optimism. We first examined the relationship between maternal depression and each of the proposed mechanisms (see Appendix Table 1). Net of the control variables, current and prior maternal depression were independently associated with all four aspects of the family environment. That is, maternal depression was positively associated with separation, environmental confusion, and parenting stress and negatively associated with relationship quality. Additionally, current and, in some cases, prior maternal depression were associated with aspects of the parent-adolescent relationship. Current and prior maternal depression were also associated with all indicators of economic wellbeing.

Table 3 considers how these proposed mechanisms explain the relationship between maternal depression and adolescent optimism. In Model 1, the baseline model (and the equivalent to Model 2 in Table 2), current maternal depression (*B* = −0.043, *p* < .05) was significantly associated with less optimism among adolescents. In Model 2, which adjusts for four indicators of the family environment, the coefficient for current maternal depression was reduced by 79% (*B* = −0.009, *n*.*s*.) and the coefficient for prior maternal depression was reduced by 70% (*B* = −0.011, *n*.*s*.). Both coefficients were statistically non-significant. In Model 3, which adjusts for the parent-adolescent relationship, the coefficients for current and prior maternal depression were reduced by 60% (*B* = −0.017, *n.s.*) and 34% (*B* = −0.023, *n.s.*), respectively. The inclusion of economic wellbeing indicators in Model 4 did not substantively reduce the magnitude of the coefficients for current and prior maternal depression.

**Table 3:** OLS regression models estimating adolescent optimism as a function of mother's depression, with mechanisms.

	Model 1				Model 2			Model 3			Model 4			Model 5		
	*Baseline*				*+ family environment*			*+ parent-adolescent relationship*			*+ economic wellbeing*			*+ all mechanisms*		
	*B*	(S.E.)			*B*	(S.E.)		*B*	(S.E.)		*B*	(S.E.)		*B*	(S.E.)	
Mother depression (reference = no depression)																
Current depression	−0.043	(0.026)	*		−0.009	(0.025)		−0.017	(0.025)		−0.043	(0.027)	*	−0.009	(0.026)	
Prior depression	−0.037	(0.022)	^		−0.011	(0.021)		−0.023	(0.021)		−0.032	(0.022)	^	−0.013	(0.021)	

*Mechanisms*																
Separation				0.015		(0.026)								0.022	(0.026)	
Parent relationship quality				0.101		(0.008)	***							0.043	(0.009)	
Environmental confusion				−0.205		(0.024)	***							−0.149	(0.025)	***
Parenting stress				−0.047		(0.014)	*							−0.029	(0.014)	
Psychological aggression								−0.054	(0.019)	**				−0.032	(0.019)	^
Physical aggression								−0.006	(0.027)					0.001	(0.027)	
Closeness to mother								0.155	(0.012)	***				0.128	(0.012)	***
Closeness to father								0.076	(0.008)	***				0.048	(0.009)	*
Parental monitoring								0.131	(0.021)	***				0.098	(0.021)	***
Employment											0.002	(0.022)		0.001	(0.021)	
Material hardship											0.017	(0.006)		0.032	(0.005)	
Household income											0.042	(0.010)	^	0.027	(0.010)	

Constant	3.100			3.546				2.242			2.914			2.644		
N	3013			3013				3013			3013			3013		

Note: Models adjust for all control variables in Model 2 of Table 2. Separation, parenting stress, material hardship, and household income are reported by mothers. Other mechanisms are reported by adolescents. Standardized coefficients presented, with standard errors in parentheses. ^ p < .10, *p < .05, **p < .01, ***p < .001.

In the final model, which includes all potential mechanisms, the relationship between maternal depression and adolescent optimism was statistically non-significant, with the mechanisms explaining 79% and 65% of the association between current and past maternal depression, respectively, and adolescent optimism. Furthermore, four mechanisms were independently associated with adolescent optimism. Environmental confusion was negatively associated with adolescent optimism (*B* = −0.149, *p* < .001). Closeness to mother (*B* = 0.128, *p* < .001), closeness to father (*B* = 0.048, *p* < .05), and parental monitoring (*B* = 0.098, *p* < .001) were positively associated with adolescent optimism. These findings suggest that the family environment and the parent-adolescent relationship, but not economic wellbeing, explain the association between maternal depression and adolescent wellbeing.

We next present results from the KHB models in Table 4, which are largely consistent with the results from the OLS regression models. For example, the KHB models highlight the importance of environmental confusion (31%, *z* = 4.73), closeness to mother (20%, *z* = 2.92), and parental monitoring (18%, *z* = 3.43) in explaining the association between current maternal depression and adolescent optimism.

**Table 4:** Results from Karlson-Holm-Breen (KHB) mediation analyses.

	% reduction	z-statistic	
Current depression			
Separation	6.8%	0.90	
Parent relationship quality	14.0%	−1.52	
Environmental confusion	31.4%	−4.73	***
Parenting stress	14.7%	−0.69	
Psychological aggression	6.0%	−1.22	
Physical aggression	−0.2%	0.09	
Closeness to mother	19.5%	−2.92	**
Closeness to father	9.9%	−0.22	
Parental monitoring	17.5%	−3.43	***
Employment	0.6%	−0.10	
Material hardship	−23.0%	0.68	
Household income	10.4%	−1.25	

Prior depression			
Separation	−6.5%	0.92	
Parent relationship quality	13.9%	−1.49	
Environmental confusion	27.5%	−3.92	***
Parenting stress	10.0%	−1.54	
Psychological aggression	4.2%	−2.00	*
Physical aggression	−0.1%	0.09	
Closeness to mother	14.3%	−2.15	*
Closeness to father	9.5%	−1.98	*
Parental monitoring	8.3%	−1.89	
Employment	30.0%	−0.11	
Material hardship	−9.2%	1.36	
Household income	5.2%	−1.10	

Note: Findings show indirect effects (percentage mediated) from the KHB-method. Percentages averaged across 20 imputed data sets. ^ p < .10, ***p < .001.

## 5 Discussion

Guided by the life course perspective (Elder, 1998), prior research on maternal depression and child outcomes (Minkovitz et al., 2005; Turney, 2011a, 2011b), and the importance of a positive future orientation for youth (Johnson et al., 2014), our study asked whether maternal depression is associated with adolescent optimism, whether this association varies by the timing of maternal depression, and whether family context factors act as mechanisms undergirding this relationship. Our results support three primary conclusions.

First, we found that current maternal depression was significantly and negatively associated with adolescent optimism, net of other contextual factors. This finding supports a major tenet of the life course perspective (Elder, 1998), that is, that family members' life courses are interconnected through the cascading repercussions of life events. We do not find support for the selection perspective, which suggests that characteristics associated with selection into maternal depression, such as socioeconomic status (Dooley et al., 2000; Lorant et al., 2003), account for the observed relationship between maternal depression and adolescent optimism. It is possible, though, that unmeasured variables—such as experiences with discrimination or broader kin relationships—may render the relationship between maternal depression and adolescent optimism spurious. Fixed effects models could account for time-stable unobserved characteristics, and future research should consider employing this analytic approach (which we were unable to do given the one-time measure of adolescent optimism).

Second, our findings regarding the timing of maternal depression were mixed. Both current and prior maternal depression were significantly associated with adolescent optimism, suggesting that the timing of maternal depression was not differentially associated with optimism. However, the association between prior maternal depression and adolescent optimism disappeared after controlling for selection. This may suggest that the repercussions of maternal depression fade over time or that maternal depression occurring at an earlier life stage does not have the same consequences for youth. The life course perspective takes “the timing of individual development and family life stage” (Bengtson, Elder, & Putney, 2011, p. 11) into account alongside broader structural and historical opportunities and events. Our models did not differentiate between timing relative to developmental age (e.g., young childhood vs. adolescence) and timing relative to distance from outcome measurement (e.g. six years vs. three months) or broader life events. To more fully examine the life course principle of timing, researchers could use a mixed age cohort to distinguish developmental stage and years from outcome measure.

Third, we found that maternal depression has repercussions for the family environment and interpersonal relationships that, in turn, shapes adolescent optimism. More specifically, we found that the family environment (especially environmental confusion) and parent-child relationships (especially adolescent relationships with mothers and reports of parental monitoring), but not economic wellbeing, are important mechanisms in the association between maternal depression and adolescent optimism. Maternal depression is associated with greater levels of environmental confusion (that is, lack of a schedule, noise, and unpredictability) and lower levels of mother-child closeness and monitoring. This, in turn, reverberates onto adolescent optimism, suggesting that protecting young people from sources of stress, strong relationships with parents, and a degree of supervision can support adolescents' positive future outlooks.

We did not find support for the proposition that economic wellbeing—measured by material hardship, income, and employment—explained the association between maternal depression and adolescent optimism. Each of these factors was associated with maternal depression, but they were not correlated with adolescent optimism. This suggests that economic wellbeing and hardship are not, by themselves, deterents of adolescents' positive future orientations. This may be due to the overriding importance of parent-child relationships for adolescents' wellbeing (Laursen & Collins, 2009) or to the prevelance of high hopes for the future in the United States more broadly (Reynolds, Stewart, MacDonald, & Sischo, 2006).

### 5.1 Limitations

Our study does have some important limitations. First, the 1-, 3-, and 5-year surveys only asked mothers to recount depressive symptoms in the year prior to the survey, although surveys were spaced more than one year apart. Thus, our measure of depression underestimates the true prevalence of this mental health problem and, accordingly, our results may be conservative because some adolescents coded as not experiencing maternal depression may have endured this in the intervening years. Second, though fathers' mental health may also be associated with adolescent optimism (Cummings et al., 2014), we were unable to consider this because only adolescents' primary caregivers (therefore, mostly mothers) were interviewed at the 15-year survey. Third, the Cronbach alpha's measure of internal consistency for adolescent optimism was low, through previous studies have found this measure to have a high level of internal consistency (Kern et al., 2016). Lastly, both the mechanisms and measure of current depression were collected in the same survey wave. In most cases, the wording of the survey ensures that time ordering is appropriate, as respondents are asked about depressive symptoms “in the past year” and about mechanisms at the time of the survey. However, parental separation, an indicator of the family environment, may have preceeded current maternal depression. Relatedly, our mechanisms may influence one another, though results from the KHB method—used to disentangle correlations between mechanisms—are consistent with our primary findings.

## 6 Conclusion

Rates of depression have been increasing in recent years, particularly with the ongoing COVID-19 pandemic. By one estimate, depression in adults increased by fourfold between 2019 and April 2020 through August 2021 (DeAngelis, 2021). Understanding the implications of this disease for those afflicted and their families is an important area of research. In this paper, we show how maternal depression reverberates onto adolescents' outlook, both in the short- and long-term. Yet we also find that maternal depression operates on adolescent outcomes through the family environment and parent-child relationships, suggesting that the repercussions of maternal depression are not inevitable and can be addressed through interventions. Our findings support attention to the family as a site where the repercussions of mental health problems manifest and the consideration of treatments that target both mothers and children (National Research Council and Institute of Medicine, 2009). Based on our findings, these interventions could focus on parent-child relationships and creating routines and structures in the home.

## Funding

Research reported in this publication was supported by the 10.13039/100009633Eunice Kennedy Shriver National Institute of Child Health and Human Development (NICHD) of the National Institutes of Health under award numbers R01HD036916, R01HD039135, and R01HD040421, as well as a consortium of private foundations. The content is solely the responsibility of the authors and does not necessarily represent the official views of the National Institutes of Health.

## Declaration of interests

The authors declare that they have no known competing financial interests or personal relationships that could have appeared to influence the work reported in this paper.

## Figures and Tables

**Table:** 

	Model 1			Model 2		
*Family Environment*						
Mother and father separated						
Current depression	0.144	(0.023)	***	0.071	(0.021)	***
Prior depression	0.127	(0.020)	***	0.071	(0.018)	***
Parent relationship quality						
Current depression	−0.148	(0.076)	***	−0.087	(0.075)	***
Prior depression	−0.137	(0.068)	***	−0.088	(0.066)	***
Environmental confusion						
Current depression	0.098	(0.018)	***	0.087	(0.020)	***
Prior depression	0.080	(0.016)	***	0.069	(0.016)	**
Parenting stress						
Current depression	0.224	(0.034)	***	0.171	(0.034)	***
Prior depression	0.141	(0.029)	***	0.081	(0.029)	***

*Parent-Adolescent Relationship*						
Psychological aggression						
Current depression	0.092	(0.024)	***	0.064	(0.025)	**
Prior depression	0.059	(0.020)	**	0.033	(0.021)	
Physical aggression						
Current depression	0.047	(0.017)	*	0.037	(0.018)	^
Prior depression	0.020	(0.015)		0.010	(0.015)	
Closeness to mother						
Current depression	−0.071	(0.040)	***	−0.063	(0.042)	**
Prior depression	−0.049	(0.034)	*	−0.035	(0.036)	^
Closeness to father						
Current depression	−0.098	(0.061)	***	−0.059	(0.060)	**
Prior depression	−0.086	(0.053)	***	−0.049	(0.052)	*
Parental monitoring						
Current depression	−0.084	(0.022)	***	−0.065	(0.023)	**
Prior depression	−0.037	(0.019)	^	−0.026	(0.020)	

*Economic Wellbeing*						
Employment						
Current depression	−0.166	(0.022)	***	−0.140	(0.023)	***
Prior depression	−0.070	(0.019)	***	−0.045	(0.020)	*
Material hardship						
Current depression	0.344	(0.085)	***	0.261	(0.085)	***
Prior depression	0.127	(0.073)	***	0.054	(0.075)	**
Household income						
Current depression	−0.166	(0.055)	***	−0.109	(0.048)	***
Prior depression	−0.077	(0.047)	***	−0.033	(0.041)	^

Note: Linear probability models estimate parent separated, parent psychological aggression, parent physical aggression, and parent employment. Ordinary least squares (OLS) regression models estimate the other outcomes. Models adjust for all control variables in Model 2 of Table 2. Standardized coefficients presented, with standard errors in parentheses. ^ p < .10, *p < .05, **p < .01, ***p < .001.
